# Characterization of a second open reading frame in genome segment 10 of bluetongue virus

**DOI:** 10.1099/jgv.0.000267

**Published:** 2015-11

**Authors:** Meredith Stewart, Alexandra Hardy, Gerald Barry, Rute Maria Pinto, Marco Caporale, Eleonora Melzi, Joseph Hughes, Aislynn Taggart, Anna Janowicz, Mariana Varela, Maxime Ratinier, Massimo Palmarini

**Affiliations:** ^1^​ MRC-University of Glasgow Centre for Virus Research, Glasgow, UK; ^2^​ Istituto Zooprofilattico Sperimentale dell'Abruzzo e Molise ‘G. Caporale’, Teramo, Italy

## Abstract

Viruses have often evolved overlapping reading frames in order to maximize their coding capacity. Until recently, the segmented dsRNA genome of viruses of the *Orbivirus* genus was thought to be monocistronic, but the identification of the bluetongue virus (BTV) NS4 protein changed this assumption. A small ORF in segment 10, overlapping the NS3 ORF in the +1 position, is maintained in more than 300 strains of the 27 different BTV serotypes and in more than 200 strains of the phylogenetically related African horse sickness virus (AHSV). In BTV, this ORF (named S10-ORF2 in this study) encodes a putative protein 50–59 residues in length and appears to be under strong positive selection. HA- or GFP-tagged versions of S10-ORF2 expressed from transfected plasmids localized within the nucleoli of transfected cells, unless a putative nucleolar localization signal was mutated. S10-ORF2 inhibited gene expression, but not RNA translation, in transient transfection reporter assays. In both mammalian and insect cells, BTV S10-ORF2 deletion mutants (BTV8ΔS10-ORF2) displayed similar replication kinetics to wt virus. *In vivo*, S10-ORF2 deletion mutants were pathogenic in mouse models of disease. Although further evidence is required for S10-ORF2 expression during infection, the data presented provide an initial characterization of this ORF.

## Introduction

Bluetongue is a haemorrhagic infectious disease of ruminants, transmitted from animal to animal via an insect vector, *Culicoides* spp., during a blood meal from viraemic hosts (Mellor *et al.*, 2009). The disease is caused by *Bluetongue virus*, a member of the *Orbivirus* genus within the family *Reoviridae*. In livestock, bluetongue is predominantly a disease of sheep resulting in severe morbidity and in some cases high mortality (Mellor *et al.*, 2009). Infection of other domestic and wild ruminants may result in clinical disease although infection is often subclinical or asymptomatic (Barratt-Boyes & MacLachlan, 1995; Caporale *et al.*, 2014; Coetzee *et al.*, 2013; Darpel *et al.*, 2007; Dercksen *et al.*, 2007; Henrich *et al.*, 2007; Maclachlan *et al.*, 2009; Mauroy *et al.*, 2008; Meyer *et al.*, 2009). Traditionally, bluetongue occurred almost exclusively in tropical and subtropical geographical areas. However, in the past 20 years there has been an expansion and incursions of several bluetongue virus (BTV) serotypes into more temperate areas such as Southern Europe, where it is now considered enzootic, and Central Europe (Elbers *et al.*, 2008a, b).

BTV possesses a dsRNA genome consisting of ten segments that encode seven structural and four non-structural proteins (Belhouchet *et al.*, 2011; Ratinier *et al.*, 2011; Roy & Noad, 2006). The virus is organized in a double-capsid structure; VP2 and VP5 make up the outer capsid of the virus and are involved in virus entry (Gouet *et al.*, 1999; Grimes *et al.*, 1998; Roy, 2008). VP2 determines the BTV serotype, of which there are 27 described to date (Huismans & Erasmus, 1981; Jenckel *et al.*, 2015; Kahlon *et al.*, 1983; Shaw *et al.*, 2013). The viral core is composed of the inner capsid proteins (VP3 and VP7), the replication complex (VP1, VP4 and VP6) and the dsRNA genome segments. Four non-structural viral proteins (NS1–NS4) are only expressed in infected cells and have essential functions in virus replication and in modulating host cell responses to virus infection (Belhouchet *et al.*, 2011; Ratinier *et al.*, 2011; Roy, 2008).

NS1 enhances viral protein synthesis and forms tubules in the cytoplasm of infected cells (Boyce *et al.*, 2012; Monastyrskaya *et al.*, 1995; Owens *et al.*, 2004). NS2 is the major component of viral inclusion bodies that are readily observed in BTV-infected cells (Butan & Tucker, 2010; Kar *et al.*, 2007; Lymperopoulos *et al.*, 2006). NS3 assists viral egress from infected cells (Beaton *et al.*, 2002; Celma & Roy, 2009; Doceul *et al.*, 2014; Vitour *et al.*, 2014) and it has also been suggested to counteract the host innate immune response by downregulating activation of the IFN-β promoter in reporter assays (Chauveau *et al.*, 2013). The most recent BTV protein to be identified was NS4. The genome of BTV and other orbiviruses was thought to be monocistronic (i.e. ten genome segments encoding ten proteins) until the recent characterization of NS4, which is encoded in the +1 reading frame of segment 9 (Belhouchet *et al.*, 2011; Firth, 2008; Ratinier *et al.*, 2011). NS4 localizes within the nucleolus of infected cells and has been shown to modulate the host IFN response by favouring viral replication *in vitro* in cells pre-treated with type I IFN (Ratinier *et al.*, 2011).

In this study, we show that the BTV genome segment 10 is also bicistronic and potentially expresses a small protein, with a putative nucleolar localization that does not, however, affect either viral replication *in vitro* or pathogenicity *in vivo* in mouse models of bluetongue.

## Results

### An additional ORF in BTV genomic segment 10 inhibits gene expression in reporter assays

**Fig. 1.**
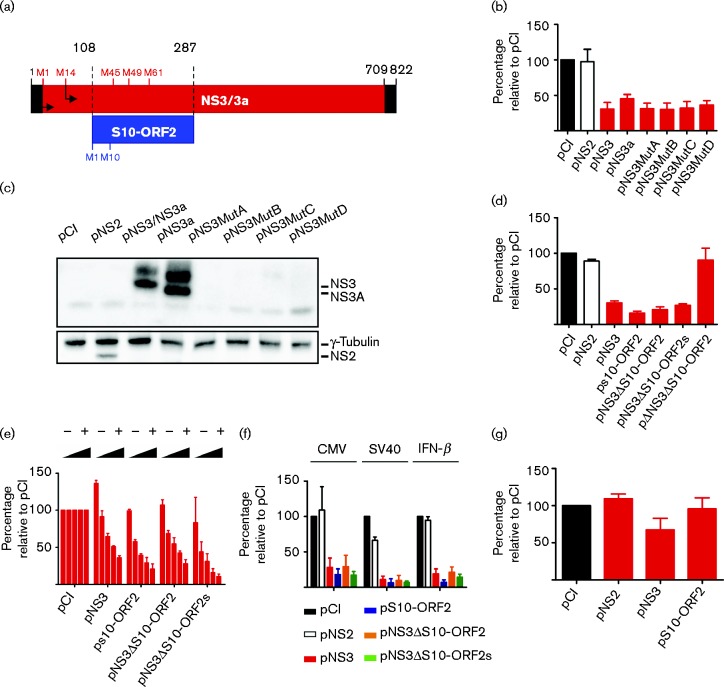
Identification of a functional ORF in BTV-8 segment 10. (a) Schematic representation of BTV-8 segment 10. The 5′ and 3′ UTRs are in black, the potential methionine (AUG) start codons within the NS3 ORF (red) prior to the first transmembrane domain are indicated above the ORF. The S10-ORF2 is indicated in blue along the potential start codons. (b) Effect of NS3 and NS3 mutants in reporter assays as described in Methods. Values (%) relative to the pCI control are plotted. (c) Western blot assessing the expression of NS3, NS3A and NS3 mutants in transfected CPT-Tert cells. (d) Effect of S10-ORF2 on luciferase activity. CPT-Tert cells were co-transfected with constructs expressing either NS2, NS3, S10-ORF2 or deletion versions of these along with pCMV-FFluc. Values (%) relative to the pCI control are plotted. (e) Dose dependent effect of S10-ORF2 and NS3 expression on luciferase activity. CPT-Tert cells were transfected as in (d) but with varying quantities of the NS3 or S10-ORF2 plasmids. (f) Influence of S10-ORF2 on reporter expression under the control of different promoters (CMV, SV40 and IFN-β). (g) Effect of S10-ORF2 and NS3 expression on *Renilla* luciferase expression from an *in vitro* transcribed mRNA. Values (%) are relative to the pCI control. (b-g) Error bars represent the sd from three independent experiments.

The starting point of this study was to investigate the role of NS3/NS3a in the general inhibition of cellular gene expression. A previous study showed that NS3, besides playing a role in virus trafficking and egress (Beaton *et al.*, 2002; Celma & Roy, 2009), was also able to inhibit the IFN-β promoter in reporter assays (Chauveau *et al.*, 2012). NS3 translation initiates from both the first AUG (M1) and the second one (M14), resulting in two isoforms of the protein, referred to as NS3 and NS3a (Fig. 1a). Here, we show using reporter assays that BTV-8 NS3 also inhibits firefly luciferase (FFLuc) expression driven by the human cytomegalovirus (CMV) immediate early promoter (pCMV-FFluc) (Fig. 1a, b). In order to assess the effects of NS3/NS3a on host cell gene expression, CPT-Tert cells were co-transfected with pCMV-FFluc together with a series of plasmids expressing NS3 (pNS3) or NS3a (pNS3a), or expressing NS3 with the first two (pNS3MutA), three (pNS3MutB), four (pNS3MutC) or five (pNS3MutD) codons encoding methionine residues mutated into codons encoding alanine residues (Fig. 1a and Methods). Reporter gene expression was significantly reduced in the presence of NS3 and all NS3 variants but not in the presence of an expression plasmid for the BTV NS2 protein (pNS2) used as control (1-way anova, *P* < 0.0001; Fig. 1b). However, by Western blotting, we could detect NS3-derived bands in lysates obtained with cells transiently transfected with pNS3 or pNS3a, but we were unable to detect any truncated forms of NS3 expressed from pNS3MutA–D (Fig. 1c).

By computational analyses of BTV-8 segment 10, we noticed the presence of an alternative reading frame in the +1 position compared with the NS3 ORF. It potentially expressed a polypeptide 59 residues in length (Fig. 1a) that we refer to from now on as ‘S10-ORF2′. We repeated the luciferase based reporter assays using constructs expressing NS3 (pNS3, which included also S10-ORF2), S10-ORF2 only (pS10-ORF2), NS3 only by interrupting S10-ORF2 (pNS3ΔS10-ORF2, pNS3ΔS10-ORF2s) or none of these proteins (pΔNS3ΔS10-ORF2) (see Methods). As shown in Fig. 1d, constructs expressing NS3, S10-ORF2, NS3ΔS10-ORF2 and NS3ΔS10-ORF2s were all able to significantly decrease reporter gene expression (1-way anova, *P* < 0.0001; Fig. 1d). No effect on luciferase activity relative to empty plasmid control was observed when cells were transfected with pΔNS3ΔS10-ORF2 or pNS2 expression constructs. Both NS3 and S10-ORF2 exerted inhibitory effects on reporter gene expression that were dose dependent and comparable to each other (Fig. 1e). In addition, the inhibitory effects of NS3 and S10-ORF2 on gene expression were not limited to reporter genes driven by the CMV immediate early promoter but they were equally efficient with other promoters such as SV40 and IFN-β (1-way anova, *P* < 0.0001) (Fig. 1f).

We also assessed whether S10-ORF2 affected protein translation by co-transfecting CPT-Tert cells with *Renilla* luciferase RNA and either pS10-ORF2, pNS3 or an empty plasmid. No inhibitory effect on *Renilla* luciferase activity was detected in the presence of BTV S10-ORF2, suggesting that this protein had little to no effect on cellular translation. We noticed, however, a statistically significant decrease of luciferase signal in pNS3 transfected cells, suggesting that the NS3 inhibitory activity may be at the translational level (1-way anova, *P* = 0.0096) (Fig. 1g).

### S10-ORF2 is under strong positive selection and is maintained in several BTV strains

**Fig. 2.**
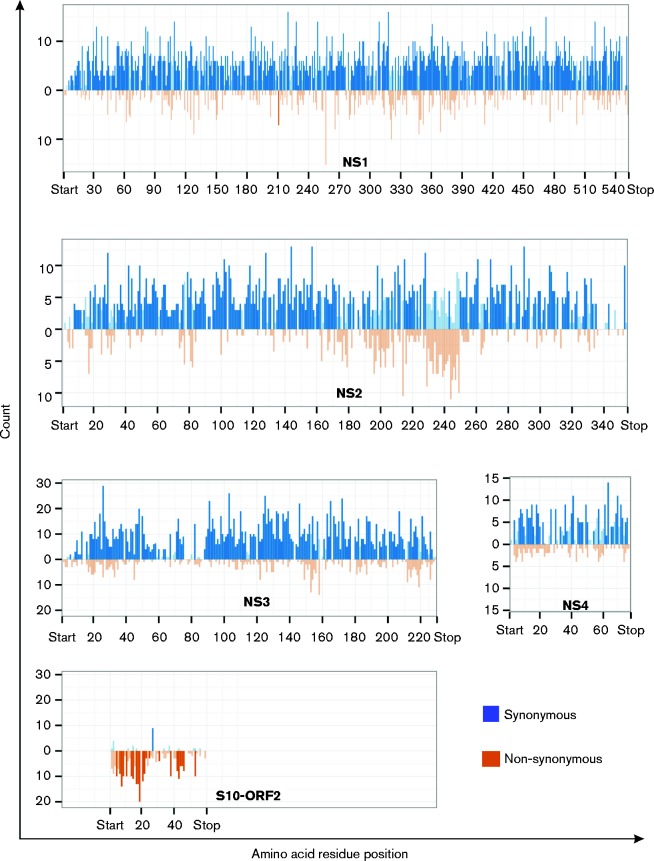
Frequency of synonymous vs non-synonymous changes in BTV non-structural proteins. Observed synonymous (blue) and non-synonymous (gold) changes determined by the SLAC method for NS1, NS2, NS3, NS4 and S10-ORF2 ORFs. Codons with significantly higher synonymous and non-synonymous changes than expected are shown in dark blue and dark gold bars, respectively. Bars with lighter colours represent values not reaching significance.

The data above suggested that segment 10 might encode a previously uncharacterized functional protein. Interestingly, S10-ORF2 was conserved in more than 300 sequences of BTV segment 10 deposited in GenBank at the time of this study. We applied different selection analyses on all the four BTV non-structural proteins and on S10-ORF2. It was apparent that BTV genes encoding non-structural proteins NS1, NS2, NS3 and NS4 are under strong purifying selection with an average dN/dS ratio below unity (Table 1). S10-ORF2, on the other hand, appears to be under strong positive selection. NS1, NS2, NS3 and NS4 have a majority of sites under negative selection while S10-ORF2 has more than a third of sites under positive selection (Fig. 2, Table 1). The corresponding gene region of S10-ORF2 in the overlapping NS3 ORF is under negative selection with the 5′-end showing evidence of neutral selection. Thus, there is greater negative selection for NS3 than for the overlapping region of S10-ORF2.

**Table 1.** Selection pressure analysis on the ORFs of non-structural proteins of bluetongue virus

Protein	Sequences analysed (*n*)	Size (aa)	dN/dS	Positively selected	Negatively selected
NS1	170	553	0.0767374	1	430
NS2	329	355	0.107094	0	224
NS3	330	230	0.0745804	0	174
NS4	159	78	0.109711	0	42
S10-ORF2	314	59	6.98739	21	1

**Fig. 3.**
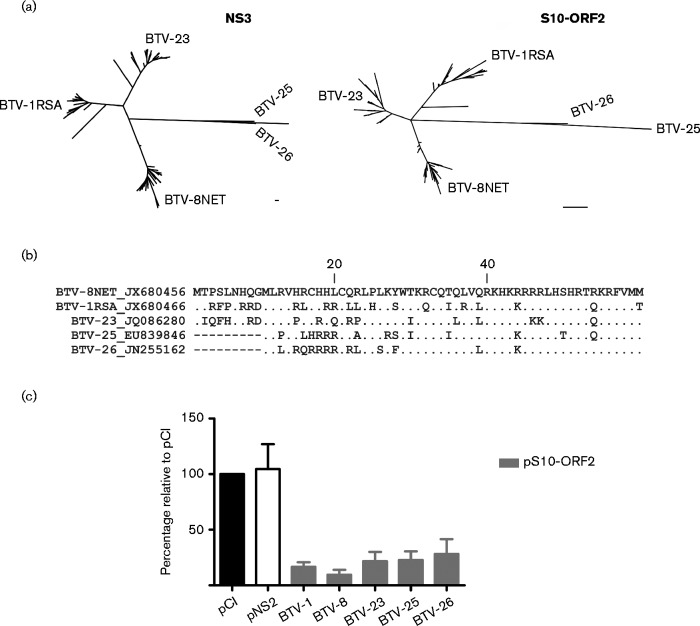
Phylogenetic analysis of NS3 and S10-ORF2 in BTV. (a) Unrooted maximum-likelihood tree of BTV NS3 (left) and S10-ORF2 (right). Bar corresponds to 10 nucleotide changes for the equivalent branch length. Bootstrap value = 1000. (b) Alignment of representative BTV strains of the four different phylogenetic groups identified. Conserved amino acids (.) and deletions (-) are indicated. (c) Reporter assays as described in Fig. 1 legend using plasmids expressing S10-ORF2 from different BTV serotypes. Values relative to the pCI-empty control (black) set at 100 % are plotted. Error bars represent the sd from three independent experiments.

Phylogenetic analysis showed that NS3 and S10-ORF2 sequences from BTV cluster into four distinct clades (Fig. 3a). As the reporter assays shown in Fig. 1 were undertaken with the S10-ORF2 from the North European strain of BTV-8, we performed reporter assays using S10-ORF2 from four other BTV strains (BTV-1, BTV-23, BTV-25 and BTV-26), representing the four distinct phylogenetic groups of NS3, to determine if there was functional conservation (Fig. 3a, b). Despite sequence variability, all the S10-ORF2 sequences tested retained the ability to inhibit gene expression in reporter assays at levels comparable to the BTV-8 S10-ORF2 (1-way anova, *P* < 0.0001; Fig. 3c).

We then assessed the orbivirus sequences deposited in GenBank for a coding region equal to or greater than 40 residues in length; we found that the S10-ORF2 ORF was maintained in African horse sickness virus (AHSV) as well as other viruses transmitted by midges and ticks, including in Kemerovo, Pata and Great Island viruses (Table 2). The S10-ORF2 ORF was not maintained in other orbiviruses such as equine encephalosis virus (EEV) or epizootic hemorrhagic disease virus (EHDV).

**Table 2.** Analysis of the +1 ORF overlapping the NS3 ORF in various orbiviruses

Orbivirus	Sequences analysed (n)	Sequences with ORF ≥ 40 codons (%)	AUG position (nt)	Number of codons
Bluetongue virus	379	100	108 or 135	59–50
African horse sickness virus	230	100	59 or 60	60–83
Epizootic hemorrhagic disease virus	22	36.3	106 or 124	40–50
Equine encephalosis virus	27	14.8	204	52
Kemerovo virus	1	100	89	62
Great Island virus	1	100	235	54
Pata virus	1	100	145	53
Umatilla virus	1	100	303	45
Heramatsu virus	1	100	243	40
Palyam virus	1	0	nd	–
Eubanangee virus	1	0	nd	–
Lebombo virus	1	0	nd	–
Orungo virus	1	0	nd	–
Wallal virus	2	0	nd	–
Warrego virus	2	0	nd	–
Corriparta virus	1	0	nd	–
Mobuck virus	1	0	nd	–
Peruvian horse sickness virus	1	0	nd	–
Sathuvachari virus	1	0	nd	–
Yunnan orbivirus	1	0	nd	–
St Croix River virus	1	0	nd	–
Tribec virus	1	0	nd	–
Wad Medani virus	1	0	nd	–

nd, None detected.

### Protein encoded by the S10-ORF2 localizes to the nucleolus in transfected cells

**Fig. 4.**
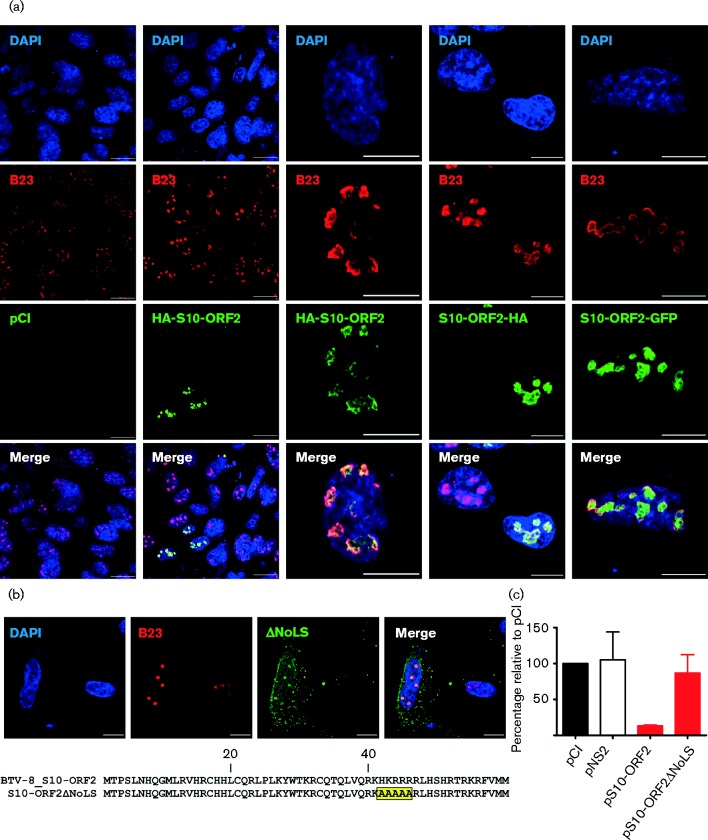
Localization of S10-ORF2 in transfected cells. (a) CPT-Tert cells were transfected with a series of constructs expressing S10-ORF2 fused at either the N- or C-terminal with HA or GFP (only at the C-terminal) or an empty pCI vector. Cells were fixed 24 h post-transfection and analysed by confocal microscopy using antibodies against the nucleolar protein B23 (in red) and HA (in green). Nuclei were stained with DAPI. Bars correspond to 20 μm for the top two rows and 10 μm for the bottom two rows. (b) Mutations of the predicted nucleolar localization signal (NoLS) within S10-ORF2. CPT-Tert cells were transfected with pS10-ORF2ΔNoLS and analysed by immunofluorescence as described above (bars, 10 μm). (c) Effect of S10-ORF2ΔNoLS in reporter assays as described in Methods. Values (%) relative to the pCI-empty control are plotted. Error bars represent the sd from three independent experiments.

We next analysed the localization of S10-ORF2 within transfected cells by confocal microscopy. Sheep CPT-Tert cells were transiently transfected with a plasmid expressing S10-ORF2 tagged with an HA epitope at its N- or C-terminus (pHA-S10-ORF2 and pS10-ORF2-HA, respectively). We observed that S10-ORF2 localized to the nucleus, and more specifically to the nucleolus, as it co-localized with the B23 nucleolar protein (Fig. 4). To ensure that the nucleolus localization of S10-ORF2 was not due to its passive diffusion through the nuclear pores (given the small size of this protein), we fused S10-ORF2 with green fluorescent protein (GFP) at its C-terminal end (pS10-ORF2-GFP). S10-ORF2–GFP also localized in the nucleolus (Fig. 4a). We then deleted a putative nucleolar localization signal (NoLS) in S10-ORF2 (Fig. 4b) by substituting amino acid residues 40–44 (HKRRR) into alanine residues (AAAAA) (Scott *et al.*, 2011). Disruption of these residues altered the localization of the resulting S10-ORF2 mutant (S10-ORF2ΔNoLS), which was observed to be dispersed widely in the cytoplasm and nucleus of the transfected cell (Fig. 4b). In addition, S10-ORF2ΔNoLS did not inhibit gene expression in reporter assays (Fig. 4c).

**Fig. 5.**
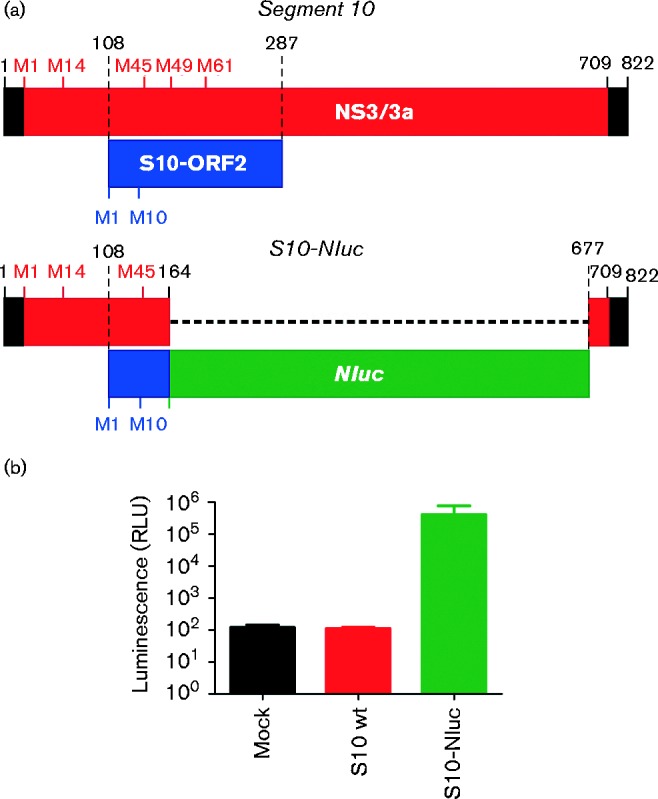
Expression of S10-ORF2 in the context of BTV genome segment 10. (a) Schematic representation of the RNA derived by *in vitro* transcription of pS10-Nluc. (b) Luciferase assays were carried out in CPT-Tert cells transfected with RNA derived from *in vitro* transcription of T7 plasmids containing either wt BTV-8 genome segment 10 (S10 wt) or S10-Nluc. Luminescence is expressed in log_10_ relative light units (RLU). Values shown are the mean of three independent experiments (*P* < 0.0001). Error bars represent the sd from three independent experiments.

A rabbit polyclonal antiserum raised against recombinant S10-ORF2 unfortunately was not able to detect expression of this protein in either BTV infected cells or in cells transfected with pS10-ORF2 or pS10-ORF2HA by either Western blotting or immunofluorescence (data not shown). We therefore derived a specific reporter construct of the size of BTV segment 10 (pS10-NLuc), in order to experimentally test whether the S10-ORF2 can be translated within the context of segment 10 (Fig. 5). This plasmid included the 5′ terminal 164 nt of segment 10 fused to the luciferase (nanoluc) gene followed by the 3′ terminal 145 nt of segment 10. pS10-Nluc has a T7 promoter and RNA transcribed *in vitro* from this plasmid is identical in length (and shares the same 5′ and 3′ termini) to wt BTV-8 Seg10 (Fig. 5a). RNA of pS10-Nluc is expected to encode the first 48 residues of NS3 and also (in the +1 ORF) the N-terminal 19 residues of S10-ORF2 fused to nanoluc. Importantly, the luciferase gene used for this construct does not contain its own start codon so that its translation is expected to initiate from either M1 or M10 of S10-ORF2. Using reporter assays, we have indeed detected luciferase expression (10^3^- to 10^4^-fold above background) in CPT-Tert cells transfected with *in vitro* transcribed RNA from pS10-NLuc (Fig. 5b), suggesting that the S10-ORF2 can be translated within the context of segment 10.

### S10-ORF2 is dispensable for BTV replication in mammalian cells and is not an IFN antagonist

**Fig. 6.**
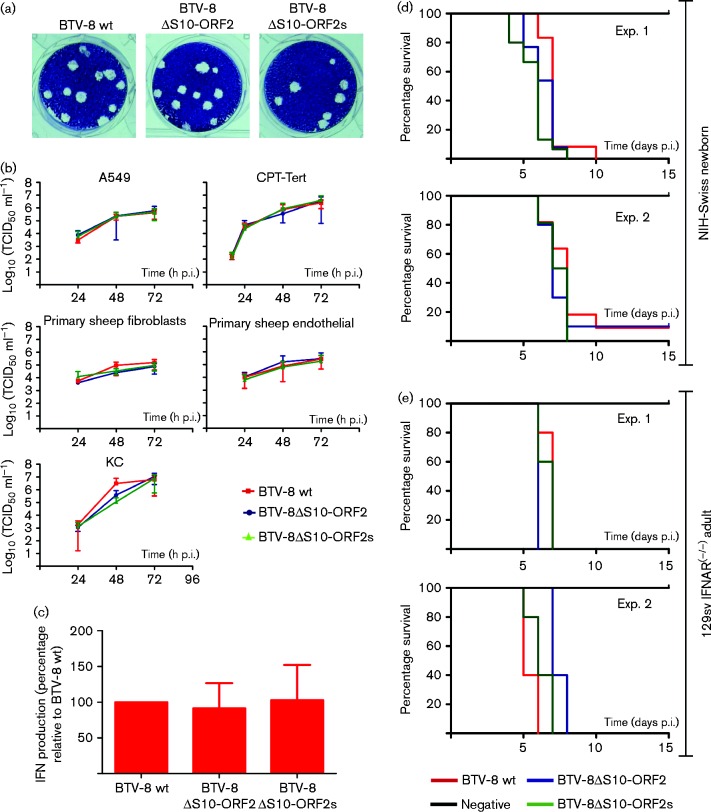
Replication kinetics and virulence of BTV8ΔS10-ORF2 viruses. (a) Plaques induced by BTV8ΔS10-ORF2, BTV8ΔS10-ORF2s and wt BTV-8 in CPT-Tert cells. (b) Replication kinetics of BTV8ΔS10-ORF2 (blue), BTV8ΔS10-ORF2s (green) and wt BTV-8 (red) in various cell types as indicated. Cells were infected with a m.o.i. of 0.001 or 0.01 as described in Methods. Supernatants were harvested at 24, 48 and 72 h post-infection and titres determined by limiting dilution in BSR cells and expressed as log_10_(TCID_50_ ml^− 1^). Each experiment was performed independently, three times, using two independently generated virus stocks. (c) Interferon response of ovEC to BTV-8 wt, BTV8ΔS10-ORF2 and BTV8ΔS10-ORF2s infection. (d) Three- to four-day-old NIH-Swiss newborn mice (*n* = 10–15) were infected intracerebrally with 300 p.f.u. of the indicated viruses. Top panel, experiment 1 (log rank test, *P* = 0.0001). Bottom panel, experiment 2 (log rank test, *P* = 0.0024). (e) Adult IFNAR^( − / − )^ (*n* = 5) were infected intraperitoneally with 100 p.f.u. of the indicated viruses. Top panel, experiment 1 (log rank test, *P* = 0.0226). Bottom panel, experiment 2 (log rank test, *P* = 0.0014). (b-c) Error bars represent the sd from three independent experiments.

In order to determine the role of S10-ORF2 during virus replication, we rescued by reverse genetics two BTV-8 S10-ORF2 deletion mutants: BTV8ΔS10-ORF2 and BTV8ΔS10-ORF2s. Both mutants induced plaques similar to those induced by wt BTV-8 (Fig. 6a). The migration profiles of the dsRNA genome of BTV8ΔS10-ORF2 and BTV8ΔS10-ORF2s were comparable to wt BTV-8 and sequence analysis revealed that there were no reversions or compensatory mutations within segment 10 (data not shown). We then carried out a series of virus replication assays in different cells, which included sheep CPT-Tert, primary ovine endothelial (ovEC) and fibroblast cells (ovFib), human A549 and culicoides KC cells. There was no significant difference in the replication kinetics of the S10-ORF2 deletion mutants in either CPT-Tert cells, which are IFN incompetent, or in ovine primary cells and the human A549 cell line. There was also no significant difference in the replication kinetics of all the viruses in the insect KC cell line. Although both BTV8ΔS10-ORF2 and BTV8ΔS10-ORF2s displayed consistently relatively lower titres than those reached by BTV-8 wt at 48 h post infection (p.i.) the difference had disappeared at 72 h p.i. (Fig. 6b).

As S10-ORF2 had the ability to shut down gene expression in reporter assays and localized to the same cellular compartment as NS4, we investigated whether it acted to suppress the IFN response during BTV infection. We carried out interferon protection assays using wt BTV-8 and the BTV-8 S10-ORF2 mutants (BTV8ΔS10-ORF2 and BTV8ΔS10-ORF2s) (Fig. 6c) and found that all viruses induced the synthesis of similar amounts of IFN in infected primary endothelial cells, suggesting that S10-ORF2 does not regulate the type I induction pathways (Fig. 6c).

### S10-ORF2 mutant viruses are lethal in experimental mouse models of disease

We next assessed whether S10-ORF2 had an influence on BTV induced pathogenicity *in vivo* using established mouse models of disease (Calvo-Pinilla *et al.*, 2009; Caporale *et al.*, 2011; Franchi *et al.*, 2008; Janowicz *et al.*, 2015). NIH-Swiss mice (3- to 4-day-old) were infected intracerebrally with 300 p.f.u. of either wt BTV-8, BTV8ΔS10-ORF2 or BTV8ΔS10-ORF2s (Fig. 6d). In parallel, adult 129sv IFNAR^( − / − )^ (*n* = 5) mice were infected intraperitoneally with 100 p.f.u. of the same viruses in each group (Fig. 6e). The experiment was performed twice using virus stocks produced independently. All mock-infected animals survived for the entire course of the experiment. As expected, in both experiments wt BTV-8 induced lethal disease in approximately 90 % of the infected mice by day 8–10 p.i. BTV8ΔS10-ORF2 and BTVΔS10-ORF2s- infected animals showed essentially the same phenotype as the mice infected with wt virus. BTVΔS10-ORF2s-infected animals appeared to have a slightly more rapid onset of disease (log rank test *P* = 0.0024 in experiment 1 and *P* = 0.0014 in experiment 2), although these small differences are of no apparent biological significance (Fig. 6d). BTV-8, BTV8ΔS10-ORF2 and BTV8ΔS10-ORF2s induced lethal disease in 100 % of 129sv IFNAR^( − / − )^ mice between days 6 and 8 p.i. in both experiments.

## Discussion

In this paper we investigated a previously uncharacterized small ORF in segment 10 of BTV, which overlaps with the major ORF encoding NS3. In the past few years, a growing body of evidence has been accumulated on the biological role of alternative reading frames in various genomes of viruses, bacteria and vertebrates (Bazzini *et al.*, 2014; Jaber & Yuan, 2013; Mohan & Atreya, 2001; Slavoff *et al.*, 2013; Storz *et al.*, 2014). Many viruses condense and conserve the maximum amount of information within their small genomes through the use of overlapping genes. Some members of the *Reoviridae* possess one or more genomic segments containing two or more ORFs (Guzmán *et al.*, 2007; Suzuki *et al.*, 1996; Voon *et al.*, 2011). The mechanism of translation initiation and expression of these gene products occurs through both leaky scanning and scanning independent mechanisms (Belli & Samuel, 1993; Firth & Brierley, 2012; Racine *et al.*, 2007). Until recently, the genome segments of orbiviruses were thought to be monocistronic. We and others have identified and characterized NS4, a non-structural protein of 77–79 amino acid residues in length modulating the IFN system (Belhouchet *et al.*, 2011; Firth, 2008; Ratinier *et al.*, 2011). Both ORFs are present in a +1 reading frame compared with the main ORFs in segment 9 and 10, respectively.

A recent bioinformatics study identified the overlapping ORF in BTV segment 10 by scanning viral sequences for regions with strong signals of synonymous constraints within the NS3 ORF (Sealfon *et al.*, 2015). These findings were based on the assumption that the rates of synonymous substitutions are lower in regions of the genome with overlapping functional elements (e.g. overlapping ORFs, secondary structures).

S10-ORF2 is conserved in more than 350 segment 10 sequences of BTV strains that have been deposited in GenBank. Further, S10-ORF2 ORF is also maintained in AHSV and a few other (but not all) orbiviruses. The conservation of an overlapping short ORF in distinct phylogenetically related viruses suggests that this is a bona fide ORF. We propose that S10-ORF2 is evolutionarily advantageous for some orbiviruses given the degree of conservation in BTV and AHSV.

S10-ORF2 appears to be the protein with the highest number of residues under positive selection among all the BTV non-structural proteins, suggesting that it may tolerate amino acid changes that do not affect its overall function and may permit adaptation to new conditions in the host cell. Sealfon and colleagues also identified a predicted putative RNA structure that could explain the bias observed between the numbers of non-synonymous and synonymous substitutions within the S10-ORF2 coding sequence (Sealfon *et al.*, 2015). We can neither rule out nor confirm the presence of important RNA secondary structures in this region from the data obtained in our *in vitro* and *in vivo* experiments. Conflicting selection pressure of overlapping proteins has previously been observed in retroviruses and papillomaviruses (Hughes & Hughes, 2005; Hughes *et al.*, 2001). Often this is characterized by high non-synonymous changes (positive selection) associated with one gene, while the second gene is associated with high rates of synonymous change (purifying or negative selection). Positive selection on the S10-ORF2 gene may be driving purifying selection on the overlapping region of NS3, or vice versa, purifying selection on NS3 may favour positive selection on S10-ORF2.

It is tempting to speculate that S10-ORF2 is a fifth non-structural protein of BTV (NS5), given its localization in the nucleolus of cells transfected with expression plasmids encoding tagged versions of this protein. Unfortunately, we have not been able to produce an antibody that recognizes this protein (by various immunoassays) even in cells transiently transfected with S10-ORF2 expression plasmids. However, our assays in cells transfected with RNA recapitulating the same length and genetic organization of BTV-8 segment 10 (and expressing the luciferase gene fused to the 5′ terminal S10-ORF2) suggest that this ORF can be translated within the context of segment 10. Definitive proof that BTV and other members of the *Orbivirus* genus express S10-ORF2 *in vivo* remains to be obtained.

In this study we have shown that S10-ORF2 was dispensable for virus replication, as BTV S10-ORF2 deletion mutants replicate as efficiently as wt BTV in both immortalized cell lines and in primary cell cultures from sheep. In addition, S10-ORF2 does not seem to provide a replication advantage to BTV in a cell line derived from *Culicoides*, nor affect viral virulence in experimental models of disease. BTVΔS10-ORF2s infection of NIH-Swiss mice resulted in moderately accelerated death of the infected animals. However, our experience with these experimental models lead us to interpret these results cautiously, even if the differences noted are statistically significant (Caporale *et al.*, 2011; Janowicz *et al.*, 2015; Ratinier *et al.*, 2011).

Interestingly, S10-ORF2 appears to localize in the nucleolus of cells transfected with expression plasmids tagged with an HA epitope or fused to GFP. Hence, it appears that S10-ORF2 has the same cellular localization as BTV NS4 and it is tempting to speculate that these two viral proteins may have a synergistic function. We established that NS4 counteracts the antiviral response of the host in cells pre-treated by type I IFN (Ratinier *et al.*, 2011) and modulates the host IFN response by inhibiting cellular transcription (Ratinier and others, unpublished data). However, despite an inhibitory effect of S10-ORF2 on the IFN-β promoter in a plasmid DNA based reporter assay, BTVS10-ORF2 deletion mutants induced amounts of type I IFN similar to those of wild-type BTV in infected cells. Indeed, in our reporter assays, S10-ORF2 appears to inhibit the activity of various promoters, suggesting that this protein could modulate cellular transcription rather than targeting the IFN-β promoter. The presence of a NoLS may suggest that BTV S10-ORF2 has evolved specific nucleolar functions. The nucleolus is a dynamic structure with multiple functions including ribosome subunit biogenesis, mediation of cell-stress responses and regulation of cell growth (Burger & Eick, 2013; Farley *et al.*, 2015). Viral proteins have been shown to interplay with the apoptotic pathway, cell cycle modulation, cellular signalling pathways, inhibition of transcription by RNA polymerase I or gene silencing (Aminev *et al.*, 2003; Emmott & Hiscox, 2009; Rawlinson & Moseley, 2015). However, in some cases, proteins can be ‘captured’ within the nucleolus by long non-coding in RNAs ‘nucleolar detention centres’ (Lam & Trinkle-Mulcahy, 2015). Hence, a protein localized in the nucleolus may not necessarily have a function related to this cellular compartment. Notably, transient expression of S10-ORF2ΔNoLS did not inhibit gene expression in reporter assays. Future studies of the interaction of S10-ORF2 with the nucleolus may provide insight into the role of this protein during infection. Ultimately, experimental infections of sheep and culicoides midges with S10-ORF2 deletion mutants might help to shed some light on the functional significance of S10-ORF2 in BTV replication and transmission.

## Methods

### Cells

HEK-293T, A549 and BSR cells (a clone of BHK21 kindly provided by Karl Conzelmann) were grown in Dulbecco's modified Eagle's medium (DMEM) supplemented with 10 % FBS. CPT-Tert cells, an immortalized sheep choroid plexus cell line, were propagated in Iscove's modified Dulbecco's medium (IMDM) supplemented with 5 % FBS (Arnaud *et al.*, 2010). Primary ovine endothelial (ovEC) cells were obtained as previously described (Varela *et al.*, 2013). Ovine primary fibroblasts were isolated from ears obtained post-mortem by standard procedures. All mammalian cell lines were cultured at 37 °C in a 5 % CO_2_ humidified atmosphere, with the exception of ovEC, which were maintained in a low oxygen incubator (37 °C, 5 % CO_2_ and 3 % O_2_). KC cells, from *Culicoides sonorensis* (Wechsler *et al.*, 1989), were grown in Schneider's insect medium supplemented with 10 % FBS and grown at 28 °C.

### Viruses

BTV-8 was rescued by reverse genetics as described previously (Boyce *et al.*, 2008; Ratinier *et al.*, 2011). BTV8ΔS10-ORF2 was rescued by reverse genetics as above with the exception that the plasmid containing segment 10 used in reverse genetics contained M1A and M10A substitutions within the S10-ORF2 ORF. BTV-8ΔS10-ORF2s was obtained as above but the S10-ORF2 ORF was interrupted by a premature stop codon in position 23, in addition to the M1A and M10A substitutions. Care was taken not to alter the NS3 ORF in BTV8ΔS10-ORF2 and BTV-8ΔS10-ORF2s.

### Plasmids

pCMV-FFluc (Palmarini *et al.*, 2000), pGL3-control (Promega) and p125-Luc (a kind gift from Takashi Fujita) (Yoneyama *et al.*, 1996) express firefly luciferase (FFLuc) under the control of the immediate early human cytomegalovirus (CMV), simian virus 40 (SV40) or the IFN-β promoters. pRL-TK (Promega) constitutively expresses *Renilla* luciferase under the control of the CMV immediate early promoter and was used to generate T7 transcripts. pFlag-CARD is an expression vector for the CARD domains of RIG-I and has been described previously (Versteeg *et al.*, 2013).

Expression plasmids (pCI, Promega) for the BTV-8 NS3 (pNS3) and S10-ORF2 (pS10-ORF2) proteins were obtained by standard procedures. Expression plasmids for NS2 of BTV-10 (NC006007), S10-ORF2 of BTV-1 (JX680466), BTV-23 (JQ086280), BTV-25 (EU839846) and BTV-26 (JN255162) were synthesized commercially (Genscript). Expression plasmids for mutated versions of the BTV-8 NS3 were obtained by site-directed mutagenesis. Plasmids expressing NS3 mutants included: pNS3a (includes the M1A mutation), pNS3MutA (M1A and M14A), pNS3MutB (M1A, M14A and M45A), pNS3MutC (M1A, M14A, M45A and M49A) and pNS3MutD (M1A, M14A M45A, M49A and M61A). To generate the pNS3ΔS10-ORF2 construct, the first two codons encoding methionine residues of the S10-ORF2 (M1 and M10) were mutated into codons encoding threonine residues. These mutations left the NS3 ORF unaltered. We also generated an additional S10-ORF2 mutant, pNS3ΔS10-ORF2s, which contained an early stop codon at amino acid residue L23 of S10-ORF2 in addition to the M1T and M10T substitutions. Finally, pΔNS3ΔS10-ORF2 contained the mutations of pNS3MutD and pNS3ΔS10-ORF2. Plasmids used for the rescue of BTV-8 by reverse genetics have been described previously (Ratinier *et al.*, 2011). Expression plasmids for BTV-8 S10-ORF2 tagged with either an HA epitope at the carboxyl- or amino-terminal domain, or GFP at the carboxyl-terminal (pS10-ORF2-HA, pHA-S10-ORF2 and pS10-ORF2-GFP, respectively) were obtained by standard cloning procedures. pHA-S10-ORF2 was mutated by site-directed mutagenesis to disrupt the predicted nucleolar localization sequence (pHA-S10-ORF2ΔNoLS). Amino acid residues in positions 42–46 (HKRRR) of the BTV-8 S10-ORF2 were all substituted into alanine residues (AAAAA) in pHA-S10-ORF2ΔNoLS.

pS10-NLuc was constructed by standard cloning procedures and includes a T7 promoter, followed by the 5′ terminal 164 nt of BTV-8 segment 10 fused to the luciferase (nanoluc) gene (missing its own start codon) and the 3′ terminal 145 nt of segment 10. The backbone of the pS10-NLuc plasmid (including the position of the T7 promoter) is identical to the plasmids used for the rescue of BTV-8 by reverse genetics (Ratinier *et al.*, 2011).

### Antibodies

Antisera against the BTV NS3 and NS2 proteins were previously described (Ratinier *et al.*, 2011). Antibodies against B23 (Sigma), γ-tubulin (Sigma) and HA (Abcam) were obtained commercially. HRP-labelled antibodies against rabbit and mouse IgGs (GE Healthcare) and secondary antibodies labelled with Alexa Fluor 488 or Alexa Fluor 594 (Invitrogen, Molecular Probes) were all purchased commercially.

### Luciferase assays

CPT-Tert cells were transfected with pCMV-FFluc (100 ng), pGL3-control (100 ng) or p125Luc (50 ng) and expression plasmids for wt or mutant forms of NS2, NS3 and S10-ORF2 (400 ng). When using p125Luc, CPT-Tert cells were concurrently transfected with 50 ng of pFlag-CARD. Dose–response experiments were carried out by transfecting 10 to 400 ng of the expression plasmids pNS3, pS10-ORF2, pNS3ΔS10-ORF2 and pNS3ΔS10-ORF2s. A similar procedure was performed to co-transfect 5 ng of *in vitro* transcribed RNA expressing *Renilla* luciferase and the indicated expression plasmids. CPT-Tert cells were transfected with an equivalent number of RNA molecules (2 × 10^11^) of either wt BTV-8 S10 or S10-NLuc generated by *in vitro* transcription. For all the experiments, cells were lysed 22 h post-transfection and firefly, *Renilla* and nanoluc luciferase activities were measured in a luminometer (20/20, Glomax) using Luciferase Assay System (Promega) as recommended by the manufacturers. The percentage of luciferase was determined by assigning the luciferase activity (expressed as relative light unit, RLU) detected in control cells (co-transfected with an empty pCI plasmid) as 100 %. To minimize the possibility that transfection efficiency affected the variability of the data, all experiments were conducted in triplicate, three independent times with two individual preparations for each plasmid.

### Virus replication curves

To compare the replication kinetics of wt BTV-8 and BTV-8 mutants, monolayers of CPT-Tert were infected at an m.o.i. of 0.001 while KC and primary fibroblast/endothelial cells were infected at an m.o.i. of 0.01, and supernatants were harvested at 24, 48 and 72 h p.i. All supernatants were clarified by low-speed centrifugation to remove cellular debris. Cell-free titres were determined by end point dilution analysis on BSR cells and expressed as log_10_(TCID_50_ ml^− 1^). Each experiment was performed three times, each time in duplicate, using two different virus stocks for each virus.

### Western blotting

Protein expression was assessed from total cell lysates by SDS PAGE and Western blotting using the various antisera indicated in the Antibodies section and as previously described (Varela *et al.*, 2013).

### Immunofluorescence and confocal microscopy

CPT-Tert cells were transfected with pHA-S10-ORF2, or pS10-ORF2-HA, pS10-ORF2-GFP or pS10-ORF2ΔNoLS. At 24 h post-transfection, cells were analysed by immunofluorescence and confocal microscopy as previously described (Caporale *et al.*, 2009).

### Interferon protection assay

Interferon protection assays were performed as previously described in OvEC cells (Varela *et al.*, 2013).

### Bioinformatic analyses

Sequences for the BTV non-structural proteins where downloaded from NCBI (29 March 2015). Sequences for each ORF were aligned using mafft (Katoh *et al.*, 2005) and duplicate sequences, sequences containing gaps or missing start/stop codons were removed from each alignment. We performed a substitution model selection in Datamonkey (Pond & Frost, 2005). The mean number of non-synonymous and synonymous substitutions per site was determined using the SLAC algorithm (Kosakovsky Pond & Frost, 2005). Site-specific selection pressures were also measured using the fixed-effect likelihood (FEL) method (Kosakovsky Pond & Frost, 2005) and the Fast Unconstrained Bayesian AppRoximation (FUBAR) method (Murrell *et al.*, 2013). For the phylogenetic analysis, 320 sequences of BTV segment 10 containing a complete NS3 ORF were extracted from GenBank using CLC Genomics Workbench software and used to estimate maximum-likelihood trees using RaxML 8.1.17 (Stamatakis, 2014). Nucleotide sequence alignments were performed using muscle (Edgar, 2004).

### *In vivo* pathogenesis studies

Animal experiments were carried out at the Istituto Zooprofilattico Sperimentale dell'Abruzzo e Molise ‘G. Caporale’ (Teramo, Italy) in accordance with local and national approved protocols regulating animal experimental use (protocol no. 11427/2012) as previously described (Caporale *et al.*, 2011). Study 1. Litters of three- to four-day-old NIH-Swiss mice (*n* = 10–15) were inoculated intracerebrally with 300 p.f.u. of wt BTV-8 and BTV-8 mutants, as indicated in the Results. A litter (*n* = 15) was inoculated with tissue culture media as a mock-infected control. Mice were killed at 14 days p.i., or earlier if showing severe clinical signs of disease. Study 2. Age-matched adult transgenic mice, deficient in the type I interferon (IFNα-β) receptor [129sv IFNAR^( − / − )^] were inoculated intraperitoneally with either 100 p.f.u. of wt BTV-8 or BTV-8 mutants, as indicated in the Results. Mice (*n* = 5) inoculated intraperitoneally with mock-infected tissue culture media were used as negative controls.

### Statistical analysis

Statistical analysis of the data was performed using GraphPad Prism.
